# *In Vivo* miRNA Decoy Screen Reveals miR-124a as a Suppressor of Melanoma Metastasis

**DOI:** 10.3389/fonc.2022.852952

**Published:** 2022-04-04

**Authors:** Rana S. Moubarak, Lisa Koetz-Ploch, Gavriel Mullokandov, Avital Gaziel, Ana de Pablos-Aragoneses, Diana Argibay, Kevin Kleffman, Elena Sokolova, Marianne Berwick, Nancy E. Thomas, Iman Osman, Brian D. Brown, Eva Hernando

**Affiliations:** ^1^ Department of Pathology, New York University (NYU) School of Medicine, New York, NY, United States; ^2^ Interdisciplinary Melanoma Cooperative Group (IMCG), New York University (NYU) Cancer Institute, New York, NY, United States; ^3^ Laura and Isaac Perlmutter Cancer Center, New York University (NYU) Langone Health, New York, NY, United States; ^4^ Department of Genetics and Genomic Sciences, Icahn School of Medicine at Mount Sinai, New York, NY, United States; ^5^ Division of Epidemiology, Biostatistics and Preventive Medicine, Department of Internal Medicine, University of New Mexico, Albuquerque, NM, United States; ^6^ Department of Dermatology, University of North Carolina, Chapel Hill, NC, United States; ^7^ Ronald O. Perelman Department of Dermatology, New York University (NYU) School of Medicine, New York, NY, United States

**Keywords:** Melanoma, metastasis, microRNA, brain metastasis, tumor suppressor

## Abstract

Melanoma is a highly prevalent cancer with an increasing incidence worldwide and high metastatic potential. Brain metastasis is a major complication of the disease, as more than 50% of metastatic melanoma patients eventually develop intracranial disease. MicroRNAs (miRNAs) have been found to play an important role in the tumorigenicity of different cancers and have potential as markers of disease outcome. Identification of relevant miRNAs has generally stemmed from miRNA profiling studies of cells or tissues, but these approaches may have missed miRNAs with relevant functions that are expressed in subfractions of cancer cells. We performed an unbiased *in vivo* screen to identify miRNAs with potential functions as metastasis suppressors using a lentiviral library of miRNA decoys. Notably, we found that a significant fraction of melanomas that metastasized to the brain carried a decoy for miR-124a, a miRNA that is highly expressed in the brain/neurons. Additional loss- and gain-of-function *in vivo* validation studies confirmed miR-124a as a suppressor of melanoma metastasis and particularly of brain metastasis. miR-124a overexpression did not inhibit tumor growth *in vivo*, underscoring that miR-124a specifically controls processes required for melanoma metastatic growth, such as seeding and growth post-extravasation. Finally, we provide proof of principle of this miRNA as a promising therapeutic agent by showing its ability to impair metastatic growth of melanoma cells seeded in distal organs. Our efforts shed light on miR-124a as an antimetastatic agent, which could be leveraged therapeutically to impair metastatic growth and improve patient survival.

## Introduction

Melanoma, together with lung and breast cancer, has a high potential to metastasize to the brain. Up to 50% of patients with stage IV melanoma have been reported to develop brain metastases at the time of their diagnosis or during the course of their illness (1). The prognosis of brain metastasis patients is poor, with a median overall survival of 17–22 weeks (2, 3), since their progression can lead to rapid neurodegeneration and a fast decline in quality of life. Despite therapeutic advances in melanoma with targeted and immune therapies (4) and the recent inclusion of brain metastasis patients in clinical trials, their prognosis remains poor. Symptomatic brain metastasis patients show a modest response to immunotherapy, while targeted therapy shows less durable intracranial activity (5, 6). A full understanding of the mechanisms controlling brain metastasis formation remains an unmet need for these patients.

Genetic alterations seem insufficient to explain melanoma metastatic behavior; thus, tumors with the same mutation profile at diagnosis can have disparate outcomes. As such, we hypothesize that non-genetic programs [i.e., epigenetic, non-coding RNA (ncRNA)] can mediate metastasis by driving a pro-metastatic transcriptional output downstream of a certain genetic makeup. Because mutations and expression profiling alone may not identify events occurring in rare cells, such as those involved in transition to metastatic phenotype, unbiased *in vivo* functional screens can help reveal alternative mechanisms.

MicroRNAs (miRNAs) are a class of 18–23-nucleotide ncRNAs, with demonstrated ability to impact multiple genes and pathways simultaneously (7). A strong body of evidence supports an important role for miRNA alterations in the tumorigenicity of multiple cancers (8, 9). miRNAs can play tumor suppressor (10) or oncogenic functions in melanoma (11–13). We and others have defined prognostic miRNA signatures for metastatic (14–16) and brain metastatic melanoma (17), shown the therapeutic potential of anti-miRNA therapies (18), and identified miRNAs that functionally contribute to various aspects of melanoma progression (19–23). Therefore, dysregulated miRNAs could regulate the complex multistep metastatic process. Despite great advances in available screen strategies using miRNA inhibition *via* Clustered Regularly Interspaced Short Palindromic Repeats (CRISPR)/Cas9 *in vitro* (24), this approach has proven challenging for efficient inhibition of miRNA biogenesis and function. In order to determine the functional role of miRNAs in metastasis *in vivo*, we performed a screen using a pooled library of miRNA Decoys (25), which revealed miR-124a as a strong suppressor of metastasis and particularly of brain metastasis. Examination of miRNA expression in human melanoma metastasis found that lower levels of miR-124a were associated with worse prognosis and with increased recurrence and brain metastasis incidence. When miR-124a was overexpressed in melanoma cells, melanoma metastasis in the brain and other organs was dramatically reduced, further demonstrating the role of miR-124a as a tumor suppressor and suggesting the potential for miR-124a mimics as a therapeutic means to control melanoma metastasis.

## Results

### *In Vivo* Unbiased miRNA Decoy Screen Identifies miR-124a as a Potential Suppressor of Melanoma Brain Metastasis

**Figure 1:**
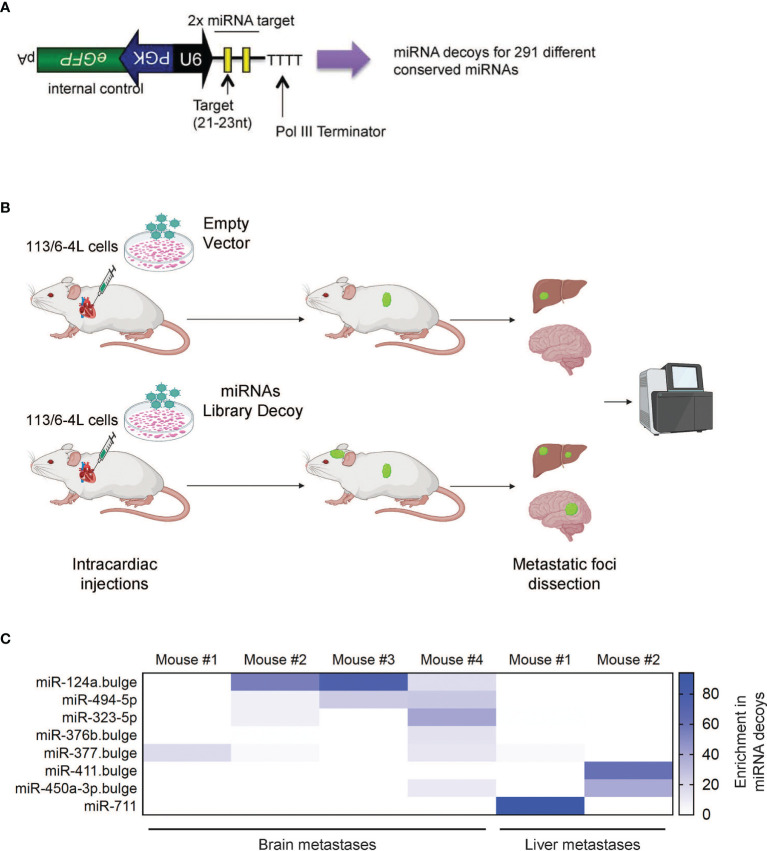
*In vivo* screen of suppressors of melanoma metastasis. **(A)** Schematic of the lentiviral vector-based miRNA decoy vector (25). miRNA decoy target sites were cloned into a lentiviral vector downstream of the U6 promoter. This vector also encodes GFP as a reporter from a separate promoter. **(B)** Schematic representation of the *in vivo* brain metastasis screen: The 113/6-4L melanoma cells transduced with control (Empty Vector) or with a lentiviral library pool of decoys targeting 291 miRNAs (miRNAs Library Decoy) were transplanted by ultrasound-guided intracardiac injection in athymic/nude female mice (Decoy Empty, n = 6; miRNA Decoy Library, n = 12). **(C)** Deep sequencing of accelerated metastatic lesions revealed miR-124a decoy as enriched (% reads for a particular Decoy over total number of reads) in brain metastases from different mice. Scale shows enrichment of specific miRNA decoys in the metastatic lesions sequenced.

We hypothesized that miRNAs could play a critical role in governing molecular mechanisms responsible for the establishment and growth of melanoma metastasis, such as chemotaxis, adhesion, migration, survival, or proliferation in the host microenvironment. First, we performed a loss-of-function *in vivo* screen for metastasis suppressors using a lentiviral library of miRNA decoy vectors targeting and inhibiting 291 conserved mouse and human miRNAs (**Figure 1A**). The efficacy of this decoy library for miRNA knockdown and loss-of-function studies has been previously demonstrated (25). As a melanoma model, we utilized 113/6-4L cells, which have been previously described to display tropism to the liver after subcutaneous injection (26). In our hands, these cells have limited ability to colonize the brain upon ultrasound-guided intracardiac injection, a well-described model of brain metastasis (27, 28). We transduced the 113/6-4L melanoma cells with the miRNA decoy Library or Empty Vector, which encodes Green Fluorescent Protein (GFP) but no miRNA decoy. Cells were transduced at a low multiplicity of infection [(MOI) 0.3] to achieve a single vector copy per cell, and then GFP+ cells were sorted to obtain a pure population of vector-expressing cells. The cells were injected into the left ventricle of athymic/nude mice, and after 2–3 months, when mice showed signs of discomfort or weight loss, they were euthanized and organs were collected for analysis. The number of brain and liver metastases was scored and GFP-positive metastatic foci were dissected, followed by deep sequencing for the detection of enriched decoys in the metastatic lesions (**Figure 1B**). To determine whether any particular miRNA decoys were enriched in the metastases, we extracted DNA from the tissues and performed deep sequencing to determine the frequency of each decoy in the library. Strikingly, out of the 291 vectors in the library, the miR-124a decoy was the dominant decoy in the metastatic lesions found in 3 out of 4 different mouse brains (**Figure 1C**). These findings suggested that inhibition of miR-124a promotes 113/6-4L melanoma metastasis to the brain.

### miR-124a Is a Potent Suppressor of Melanoma Metastasis

**Figure 2:**
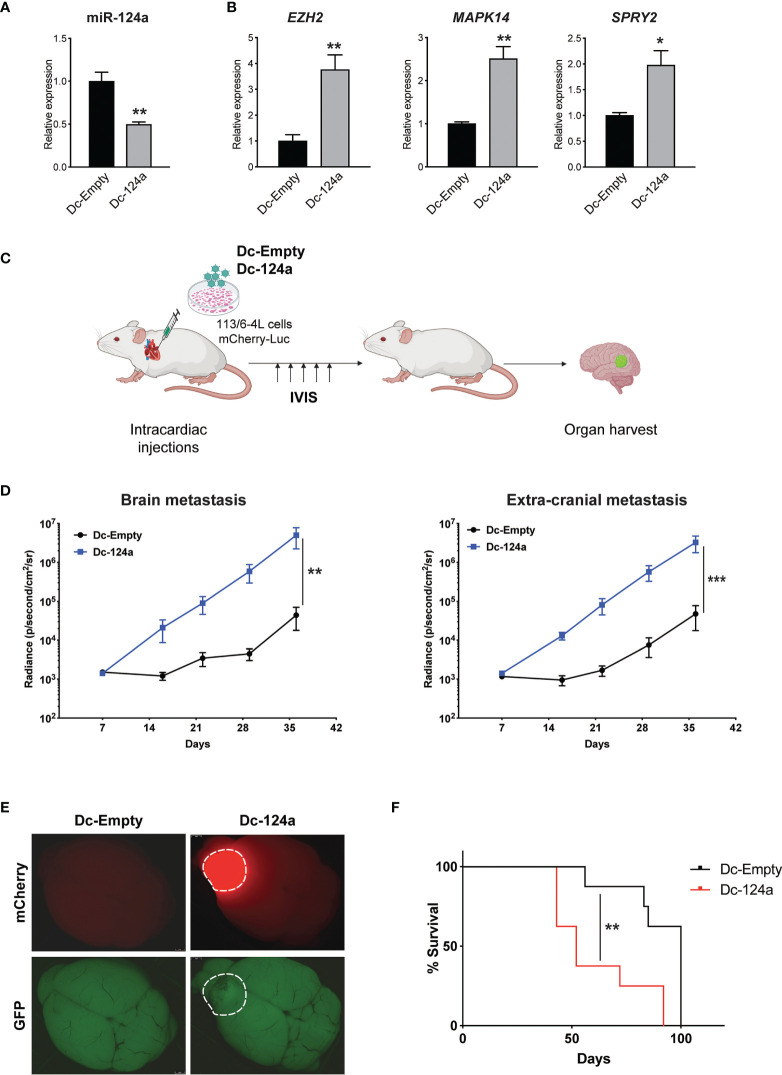
miR-124a silencing enhances melanoma metastasis. **(A)** Relative expression of miR-124a (unpaired t test; **p = 0.003) and **(B)** its targets was measured by quantitative RT-PCR after lentiviral infection of 113/6-4L with mCherry-Luciferase and Dc-Scr or Dc-124a tagged with a GFP reporter (unpaired t test; *EZH2* **p = 0.0019, *MAPK14* **p = 0.0059, and *SPRY2* *p = 0.0189). **(C)** Schematic representation of the *in vivo* metastasis model in athymic/nude female mice (n = 8 per group). **(D)** miR-124a silencing significantly accelerates both brain (**p = 0.0069) and extracranial metastasis (***p = 0.001) burden, as measured by *in vivo* bioluminescence imaging using IVIS (Mann–Whitney test). **(E)** Representative brain images at termination were obtained using a dissecting scope (Leica). **(F)** Overall survival is significantly decreased upon miR-124a silencing. Mice were humanely euthanized once they present symptoms of distress or >20% of weight loss. The experiment was terminated when mice from the Dc-Scr group that remained alive at day 100 were euthanized (Mantel–Cox test; **p = 0.0055).

miR-124a was initially described as a neuron-specific miRNA and is not detected at appreciable levels in cells outside the nervous system (29, 30). It has an important role in neuronal differentiation (31) and was subsequently shown to function as a tumor suppressor gene in the brain and found to be lost in glioblastoma (32). However, a role for miR-124a in melanoma metastasis has not been described, though it was intriguing that its inhibition was associated with brain metastasis. To validate miR-124a’s potential function as a metastasis suppressor, we transduced 113/6-4L melanoma cells with lentiviral vectors carrying mCherry-luciferase and a GFP-tagged single decoy against miR-124a (Dc-124a) or a scrambled decoy control (Dc-Scr). We confirmed that the miR-124a decoy efficiently inhibited miR-124, as miR-124a levels were reduced (**Figure 2A**), an indicator of decoy activity (33), and established targets of miR-124a EZH2 (34), MAPK14 (35), and SPRY2 (36) were significantly upregulated (**Figure 2B**). Nude mice were inoculated with Dc-124a- or Dc-Scr-transduced melanoma cells in the left heart ventricle and monitored by luminescence imaging throughout the experiment (**Figure 2C**). The group inoculated with Dc-124a-expressing cells displayed a significantly accelerated brain and extracranial metastasis burden relative to control (**p = 0.0069 and ***p = 0.001, respectively) (**Figures 2D, E** **)**. Accordingly, miR-124a knockdown significantly shortened mouse survival in this model (**p = 0.0055) (**Figure 2F**). These data validate the findings of our positive selection screen, confirming miR-124a as a metastasis suppressor miRNA.

**Figure 3:**
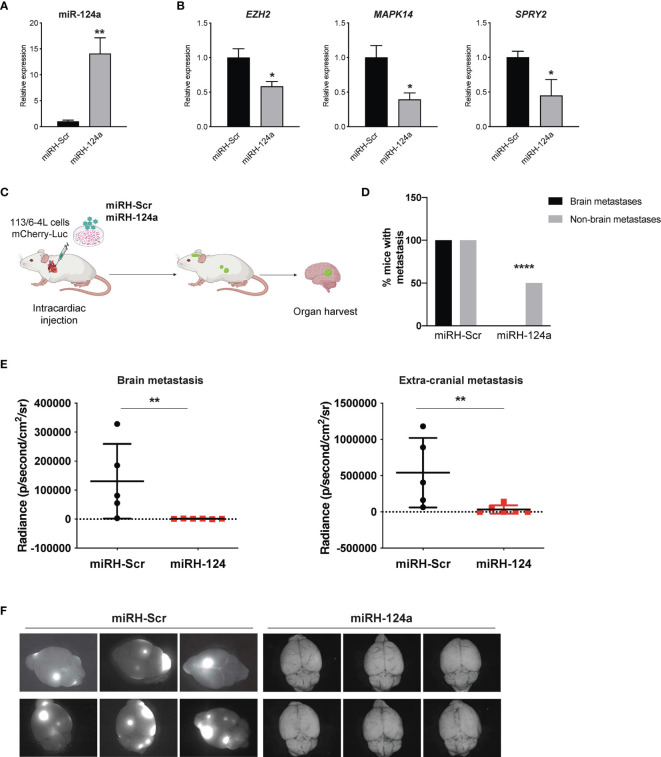
miR-124a constitutive overexpression suppresses metastasis and particularly brain metastasis. The 113/6-4L-mCherry-Luciferase cells were transduced with miRH-Scr or miRH-124a tagged with a GFP reporter. **(A)** Efficient miRNA-124a overexpression (unpaired t test; **p = 0.0083) and **(B)** consequent repression of its targets were measured by quantitative RT-PCR (unpaired t test; *EZH2* *p = 0.05, *MAPK14* *p = 0.02, and *SPRY2* *p = 0.038). **(C)** Schematic representation of the *in vivo* metastasis model using cells generated in **(A)** in nude female mice (n = 6 per group). **(D)** Incidence of brain and extracranial metastases in both groups is shown. Extracranial metastases refer to lung and/or ovary metastases (two-sided chi-square test, ****p < 0.0001). **(E)** miR-124a overexpression significantly inhibits both brain (**p = 0.0043) and extracranial metastasis (**p = 0.0087) burden, as measured by *in vivo* bioluminescence imaging using IVIS at experiment termination (Mann–Whitney test). **(F)** Representative GFP fluorescence pictures of brain metastasis in mice from both groups are shown (Leica).

To further validate these findings, we conducted a gain-of-function approach. We transduced 113/6-4L cells with a lentivirus expressing mCherry-luciferase, together with a lentivirus overexpressing miR-124a coupled to GFP (miRH-124a) or its scrambled control (miRH-Scr). MiR-124a upregulation (**Figure 3A**) effectively reduced the expression of its established targets EZH2, MAPK14, and SPRY2 (**Figure 3B**). miRH-124a- or miRH-Scr-transduced cells were inoculated in the left ventricles of nude mice using ultrasound-guided intracardiac injection (**Figure 3C**). Mice were euthanized 5 weeks post-injection when some started to display signs of discomfort. In agreement with its loss-of-function effects, miR-124a overexpression significantly suppressed brain and extracranial metastases to lungs and ovaries (two-sided chi-square test, ****p < 0.0001; **Figure 3D**), as measured by bioluminescence at experiment termination (brain metastasis, **p = 0.0043; extracranial metastasis, **p = 0.0087; **Figure 3E**). Remarkably, while all mice injected with miRH-Scr-transduced cells developed brain metastases, none of the mice injected with miRH-124a-overexpressing cells developed any brain metastasis (**Figures 3D–F**). We conclude that miR-124a overexpression abolishes brain metastasis formation and significantly inhibits melanoma metastasis to other organs.

To determine if miR-124a was suppressing growth, rather than metastasis specially, we examined the effect of either constitutive or inducible miR-124a overexpression in relevant xenograft models. We subcloned the miR-124a precursor and its Scrambled control (Scr) into the TRIPZ™ inducible lentiviral vector (Dharmacon). This vector is configured for the expression of shRNAs and miRNAs of interest, together with the turboRFP (tRFP) reporter, in the presence of doxycycline (Tet-On configuration). Importantly, in this construct, miRNA expression and tRFP expression are coupled, as both arise from the same promoter, in a doxycycline-dependent manner.

**Figure 4:**
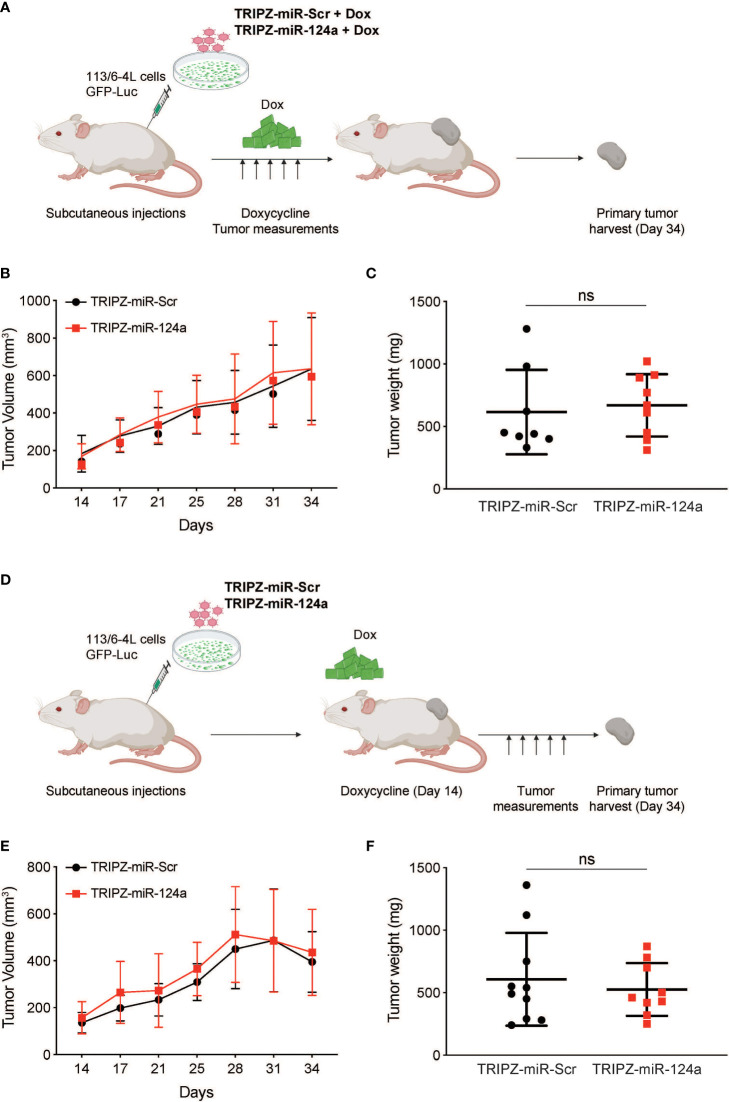
miR-124a overexpression does not inhibit subcutaneous tumor formation nor established tumor growth. **(A)** Schematic representation of *in vivo* tumor growth model: The 113/6-4L -GFP-Luciferase cells were stably transduced with doxycycline-inducible control TRIPZ-miR-Scr or TRIPZ-miR-124a tagged with a tRFP reporter and injected with Matrigel (1:1) into the flank of NSG female mice. Cells were treated with doxycycline (2 µg/ml) prior to injection, and mice were fed doxycycline chow since the intracardiac inoculation. **(B)** Constitutive miR-124a overexpression in 113/6-4L cells did not affect tumor volume **(C)** nor weight at termination of the experiment (Day 34). **(D)** Schematic representation of *in vivo* growth after implantation: The 113/6-4L -GFP-Luciferase cells were stably transduced with doxycycline-inducible control TRIPZ-miR-Scr or TRIPZ-miR-124a tagged with a tRFP reporter and injected with Matrigel (1:1) into the flank of NOG/SCID female mice. Once tumors were palpable (Day 14 post-injection), mice were fed doxycycline chow (200 mg/kg). **(E)** Inducible miR-124a overexpression in established tumors did not affect tumor volume **(F)** nor weight at termination of the experiment (Day 34). ns, non significant.

Melanoma cells were transduced with a GFP-luciferase–expressing construct, followed by TRIPZ-Scr/miR-124a lentiviral particles, and were injected subcutaneously in the flanks of immune-compromised NOD/Scid/IL2γRnull (NSG) mice. Cells were treated with doxycycline prior to inoculation, and mice were fed doxycycline-containing food pellets immediately for constitutive miR124a overexpression (**Figure 4A**). Once palpable, tumor growth was regularly measured by caliper. In another set of experiments, mice were fed doxycycline-containing food pellets starting 14 days after inoculation, when tumors became palpable (**Figure 4D**). Comparison of tumor growth found that neither miR-124a constitutive (**Figures 4B, C**) nor inducible overexpression after tumors’ engraftment (**Figures 4E, F**) affected tumor growth. This indicates that miR-124a specifically suppresses melanoma metastatic progression without affecting primary tumor growth.

### miR-124a Overexpression Impairs the Growth of Seeded Metastases

**Figure 5:**
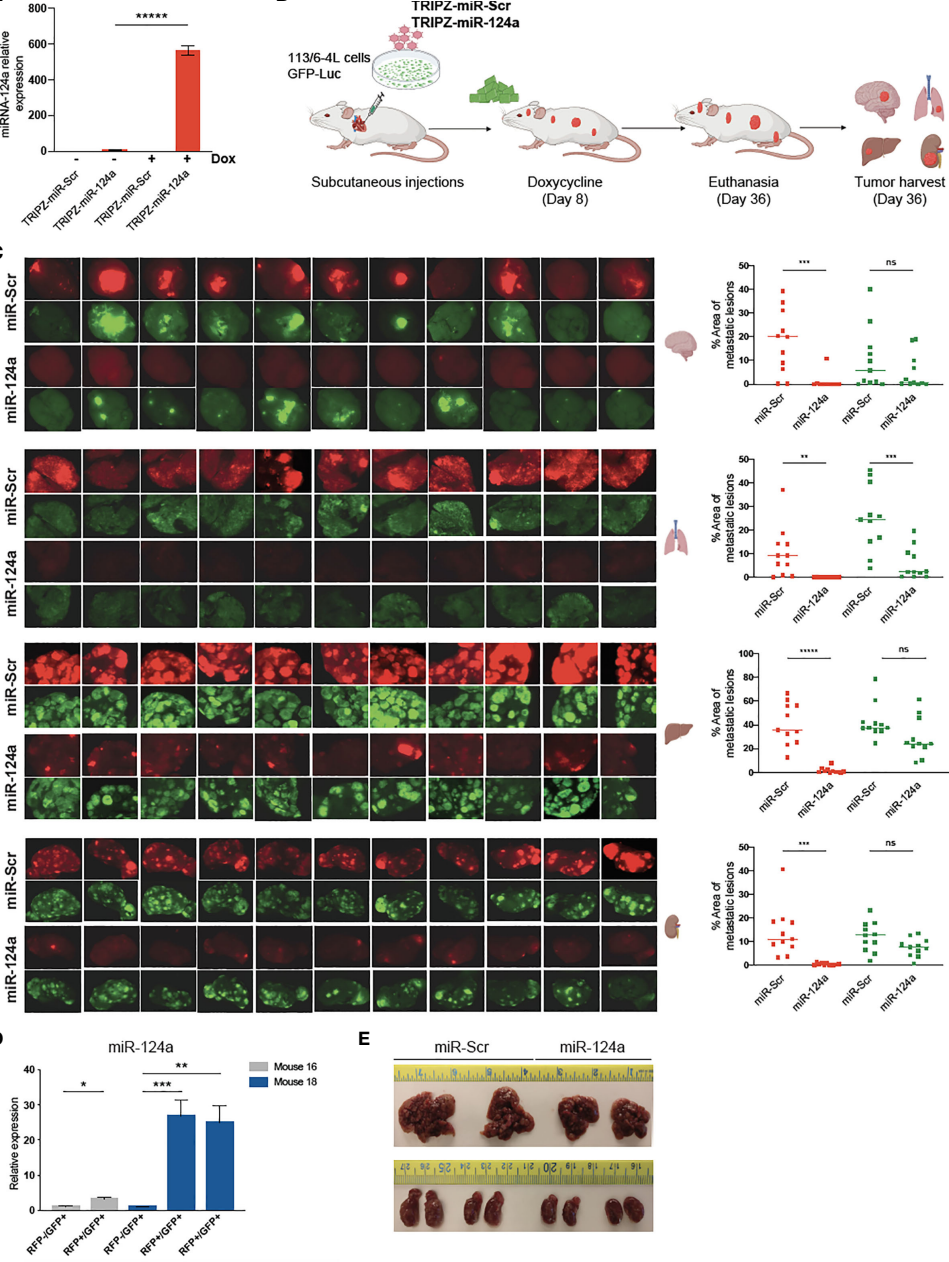
miR-124a overexpression inhibits established metastases. **(A)** The 113/6-4L melanoma cells were transduced with GFP-Luciferase followed by TRIPZ-Scr or TRIPZ-miR-124a lentiviral infections. Efficient miRNA-124 overexpression after doxycycline treatment *in vitro* was assessed by RT-qPCR (unpaired t test; *****p = 0.000005). **(B)** Schematic representation of *in vivo* metastasis assay with inducible overexpression of miR-124a: The 113/6-4L-GFP-Luciferase cells transduced with doxycycline-inducible control (TRIPZ-miR-Scr) or (TRIPZ-miR-124a) tagged with a tRFP reporter were selected with puromycin, and ultrasound-guided intracardiac injection was performed in NSG mice (n = 11 per group). Mice were initiated on doxycycline (200 mg/kg) chow 8 days post-injection. **(C)** RFP and GFP fluorescence images of brain, lung, liver, and kidneys in all mice are shown, and the areas of metastatic lesions were plotted using ImageJ (red dots: RFP+ metastases; green dots: GFP+ metastases). Unpaired t test was used for statistical significance of differences in brain (***p = 0.0006 for RFP+ metastases in miR124a vs. miR-Scr group; ns, p = 0.274 for GFP+ in miR124a vs. miR-Scr group), lung (**p = 0.0041 for RFP+ metastases in miR124a vs. miR-Scr group; ***p = 0.00056 for GFP+ in miR124a vs. miR-Scr group), liver (*****p < 0.000001 for RFP+ metastases in miR124a vs. miR-Scr group; ns, p = 0.056 for GFP+ in miR124a vs. miR-Scr group), and kidney (***p = 0.00033 for RFP+ metastases in miR124a vs. miR-Scr group; ns, p = 0.058 for GFP+ in miR124a vs. miR-Scr group) metastases. **(D)** Expression of miR-124a in RFP-/GFP+ and RFP+/GFP+ liver metastatic foci dissected from two representative mice (#16 and #18) from the miR124a group is measured by quantitative RT-qPCR (unpaired t test; *p = 0.018 for mouse 16 and ***p = 0.0011 and **p = 0.005 for mouse 18). **(E)** Representative images of the liver and kidney sizes of two mice from both groups are shown. ns, non significant.

We postulated that miR-124a overexpression in already seeded metastatic cells might impair further growth. To test this hypothesis, we aimed to conditionally induce miR-124a in melanoma cells after reaching distal organs. Melanoma cells were transduced with GFP-Luciferase followed by TRIPZ-Scr or TRIPZ-miR-124a lentiviral infections. Efficient miRNA-124a overexpression after doxycycline treatment *in vitro* was assessed by qRT-PCR (**Figure 5A**). Subsequently, puromycin-selected cells cultured in tetracycline-free serum were inoculated in the left ventricle of NSG mice. We have found in previous studies that most melanoma cells have already extravasated from the vasculature and colonized the brain microenvironment by day 7 after intracardiac injection in mice (Kleffman et al., Biorxiv). We thus started feeding mice with doxycycline-containing chow at day 8 after cell inoculation, aiming for efficient miR-124a induction 2–3 days later. The experiment was terminated 36 days after inoculation, when some mice showed signs of discomfort (**Figure 5B**). Harvested organs (brain, liver, lung, and kidneys) were imaged using a dissecting scope to detect both GFP+ (which marks all melanoma cells) and RFP+ (which marks miR-Scr- or miR-124a-expressing cells) (**Figure 5B**). In the group injected with miR-Scr-transduced cells, most mice developed brain metastases, and the GFP and RFP signals vastly overlapped (**Figure 5C**, upper brain panels). In the miR-124a-transduced group, however, RFP+ lesions were found absent from all harvested brains, with the exception of a small lesion in one mouse (**Figure 5C**; brain panels, eighth counting from left). In the liver and kidney, we found a similar suppression of RFP+ lesions, further supporting a negative selection of cells carrying miR-124a overexpression. These results strongly suggest that miR-124a expression is deleterious for metastases, and only those cells that escape miR-124a induction, either by insufficient doxycycline levels or by compensatory adaptations, are able to successfully colonize the brain. To prove this, we tested if RFP fluorescence was a faithful reporter of miR-124a overexpression. In contrast to the brain, several liver metastatic lesions were GFP+/RFP+ in the mice injected with miR-124a-transduced cells. We carefully dissected RFP-/GFP+ and RFP+/GFP+ liver metastatic foci from 2 different mice of the miR-124a-overexpressing group and assessed miR-124a expression by qRT-PCR. We found that RFP+/GFP+ lesions have significantly higher miR-124a expression than RFP-/GFP+ lesions from the same mice (**Figure 5D**). While miR-124a-overexpressing lesions were still present in liver and kidneys (**Figure 5C**), metastasis burden and organ size were reduced in mice injected with miR-124a-overexpressing cells than those instilled with miR-Scr-transduced cells (**Figure 5E**). Taken together, our data indicate that miR-124a acts as a potent barrier against metastatic growth, and that cells expressing miR-124a are negatively selected in general and even more so in the brain parenchyma, supporting its value as an anti-metastatic miRNA.

### miR-124a Levels in Primary Melanoma Correlate With Recurrence, Brain Metastasis, and Brain Metastasis-Free Survival

**Figure 6:**
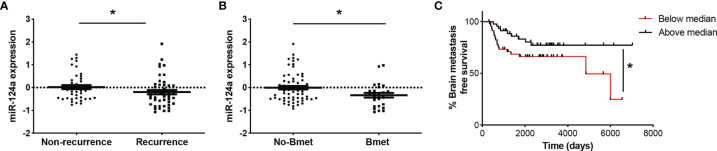
Low miR-124a levels in primary melanoma correlate with recurrence, brain metastasis recurrence, and decreased brain metastasis-free survival. Primary human melanoma samples were collected at the time of surgery from 92 patients enrolled in the NYU Melanoma Program (IMCG) database. miRNA expression profiling of FFPE-extracted RNAs from primary melanomas was performed by Exiqon, Inc., using miRCURY™ LNA arrays (Exiqon, Denmark) (20). **(A)** Relative miRNA-124 expression was compared between primary tumors based on their recurrence status (Mann–Whitney test; *p = 0.047). **(B)** Relative miRNA-124 expression in primary tumors that eventually metastasized to the brain (Bmet) vs. primary tumors that did not metastasize or metastasized to other organs (No-Bmet) (Mann–Whitney test; *p = 0.02). **(C)** Brain metastasis-free survival analysis shows that low miR-124a correlates with increased brain metastasis incidence (Breslow–Wilcoxon test; *p = 0.05). Low and high miR-124a levels are relative to the median expression.

miRNA profiling of 92 human primary melanoma specimens (stages I and II, GSE62372) (20) revealed higher expression of miR-124a in non-recurrent (n = 44) vs. recurrent (n = 48) primary melanoma samples (p = 0.047; **Figure 6A**). Moreover, primary tumors that eventually metastasized to the brain (n = 26) displayed lower miR-124a levels compared to those that did not (n = 66) (p = 0.02; **Figure 6B**). Finally, low miR-124a levels associate with decreased brain metastasis-free survival (p = 0.05; **Figure 6C**). These data further support the clinical relevance of miR-124a as a negative regulator of metastasis in general and of brain metastasis in particular.

## Discussion

Melanomas are highly metastatic tumors, thus representing a pertinent model to study metastatic progression. However, genetic alterations have been deemed insufficient to explain melanoma metastatic potential. While melanoma cells display a high propensity to translocate from primary to distant metastatic sites, the colonization of a host microenvironment can represent a bottleneck for metastasis (37). Only those cells that undergo molecular and cellular changes befitting their new environment will be able to adapt and survive in distant organs (38–41). Brain metastases grow in part by co-opting existing blood vessels and astrocytes (42) and are the most common types of brain tumor leading to high morbidity and mortality. In the United States, approximately 150,000 cases of brain metastases are diagnosed every year (43). A variety of primary tumors such as breast, lung, and melanoma can develop brain metastasis through complex mechanisms during the multiple-step metastatic process (44).

Metastatic cells can harbor aberrant signaling or express dysregulated ncRNAs to promote their motility and survival. We and others have shown that miRNAs and circular RNAs (7, 45–48) play important roles in these processes. miRNAs regulate gene expression in multiple ways, such as translational repression, mRNA cleavage, and mRNA decay. Roles for miR-146a in suppressing (49) and for miR-210 in promoting (50) melanoma brain metastasis have been reported.

In this study, we undertook an unbiased approach to investigate the ability of miRNAs to suppress the metastatic fitness of malignant cells using an *in vivo* screen with positive selection. A decoy of miRNA-124a appeared consistently enriched in multiple brain metastasis lesions, suggesting that this miRNA could be a strong suppressor of melanoma brain metastasis (**Figure 1**). This result was unexpected because miR-124a is a miRNA the expression of which is associated with the brain and not melanoma. We further validated miR-124a as able to impair both brain and extracranial metastases (**Figures 2**, **3**). Future studies should be conducted in additional cell lines to further strengthen the general applicability of our findings to melanomas with various genetic backgrounds. It has been shown that melanin synthesis can affect melanoma behavior (51, 52). We did not observe noticeable changes in pigmentation upon manipulation of miR-124 levels *in vivo* in both primary tumors and metastatic lesions. The role of melanin synthesis in metastatic progression merits further investigation and should be formally examined. An antitumoral role for miR-124a, the most brain-abundant miRNA (53), has been previously reported in glioblastoma (54, 55). It can be packaged in extracellular vesicles, and released miRNA-124a has been found to regulate the glutamate transporter 1 in astrocytes, thus influencing astrocyte-mediated regulation of neurons in a feedback mechanism (56, 57). These observations raise the paradox of a potential crosstalk between metastatic melanoma cells that silence miRNA-124a for adaptation to a miRNA-124a-abundant brain microenvironment. The precise understanding of this crosstalk could reveal pathways that could be targeted to prevent melanoma adaptation in the brain. miRNA-124a has been shown to function as a tumor suppressor by targeting Receptor for Activated Protein C Kinase (RACK1) in cutaneous melanoma (58) and the histone methyltransferase Enhancer of Zest Homolog 2 (EZH2) in uveal melanoma (59). The functional role of EZH2 in promoting melanoma metastasis has been well established (60), suggesting its potential contribution to miRNA-124a metastasis suppressive role reported here.

Our findings suggest that miRNA-124a could serve as a therapeutic target for the treatment of brain and extracranial melanoma metastasis, since its delivery using an inducible system impairs the growth of seeded melanoma cells in the brain (**Figure 5**). This would be particularly impactful against melanoma brain metastasis, given that many patients are non-responsive or develop resistance to checkpoint blockade, the current standard of care for metastatic melanoma. The use of ncRNAs for therapeutic purposes remains an area of active research. A current limitation of miRNA delivery is their tendency to accumulate in the liver with potential toxicity and limited access to other tissues (61). However, new strategies and developments may overcome the biological challenges of miRNA delivery and allow exploiting their therapeutic potential. Recent advances in drug delivery systems using lipid nanoparticles have expedited the preclinical development of mRNA therapeutics (62–64), providing the basis for Food and Drug Administration (FDA)-approved coronavirus disease 2019 (COVID-19) mRNA vaccines (65–67). Additionally, novel chemical modifications for increased mRNA stability utilized for these vaccines could be exploited for improved miRNA stability (68).

Finally, we have shown that miR-124a expression in primary melanomas may be able to predict recurrence and brain metastasis incidence (**Figure 6**). Therefore, miR-124a holds promise as a biomarker for selection of early-diagnosed patients who would highly benefit from increased surveillance or recently approved adjuvant therapies (69–71).

## Methods

### Cell Culture

Cell lines were grown at 37°C in an atmosphere of 5% CO_2_. HEK293T cells purchased from American Type Culture Collection (ATCC) were maintained in Dulbecco's Modified Eagle's Medium (DMEM) medium (*Invitro*gen) containing 10% (v/v) FBS and 1% (v/v) penicillin/streptomycin. The 113/6-4L cells (gift of Dr. Robert Kerbel, University of Toronto) were cultured in DMEM (*Invitro*gen) containing 10% (v/v) FBS (Corning) and 1% (v/v) penicillin/streptomycin (*Invitro*gen). The 113/6-4L is a metastatic variant of the WM239A human melanoma cell line (26). Cells were maintained in culture for no more than 20 passages and were routinely tested for mycoplasma contamination (Universal Mycoplasma Detection kit, ATCC).

### Viral Production

In this study, 3 × 10^6^ HEK293T cells were seeded per 10-cm tissue culture dish and co-transfected with lentiviral expression constructs (12 μg), viral packaging plasmid (pSPAX2, 8 μg), and viral envelope plasmid (pMD2.G, 4 μg) using Lipofectamine2000 (Invitrogen), following manufacturer’s recommendations. Viral supernatant was collected 48 h post-transfection, filtered through 0.45-μm filters, and stored at -80°C for long-term storage.

### Viral Transduction

Target cells were seeded, incubated overnight prior to infection, and transduced at 30% of cell confluence. Medium was replaced with 1:4 diluted viral supernatant and 4 μg/ml Polybrene (EMD Millipore) and incubated for 6 h, followed by replacement with growth medium. Cells were checked for fluorescent protein expression, or drug selection agents were added on subsequent days to ensure pure populations of transduced cells. For viral transduction of melanoma cells with the Decoy library, cells were transduced at an MOI of 0.3 to achieve a single vector copy per cell, resulting in transduction of 30% of cells plated in 150-mm^2^ dishes. The percentage of GFP-positive transduced cells and their viability were assessed by flow cytometry 3 days after transduction (BD LSR II, BD Biosciences).

### RNA Extraction From Cultured Cells and Metastatic Foci

RNA was extracted using the RNeasy mini kit (Qiagen) following manufacturer’s recommendations. Eluted RNA was quantified by Nanodrop 2000 or Qubit (Thermo Fisher) following manufacturer’s recommendations and stored at -80°C. When indicated, metastatic foci with RFP and/or GFP signal were dissected using a dissecting fluorescent scope (Leica). Samples were immediately frozen in TRIzol buffer prior to RNA extraction.

### miRNA Quantification by RT-qPCR

Reverse transcription (RT) was carried out using TaqMan MicroRNA Reverse Transcription Kit in the presence of RNase inhibitor (Applied Biosystems). Briefly, 25 ng of input RNA was reverse transcribed by stem-loop method following manufacturer’s recommendations. To determine the levels of mature miRNA-124a, quantitative Real time PCR (qPCR) was performed following cDNA generation. Briefly, 1 µl of cDNA was used as template in a 10-µl qPCR reaction by adding TaqMan Universal Master Mix II, no UNG (Applied Biosystems), and predesigned TaqMan MicroRNA Assays PCR primers and probes (FAM dye-labeled) for target miRNA-124a (ID: 001182). RNU44 small RNA was used as an endogenous reference gene (ID: 001094). All reactions were performed in triplicate using Biorad CFX 384 or ABI StepOne Plus real-time cyclers following manufacturer’s recommendations.

### mRNA Quantification by RT-qPCR

In this study, 1,000 ng of RNA was reverse transcribed using Applied Biosciences Taqman RT kit (Applied Biosystems, Thermo Fisher) following manufacturer’s recommendations. cDNA was diluted with nuclease-free H_2_O prior to use in qPCR reactions. Glyceraldehyde 3-phosphate dehydrogenase (GAPDH) was used as endogenous reference gene in RT-qPCR experiments. Biorad CFX 384 or CFX96 real-time cyclers were used with the following 2-step cycling parameters: 10 min at 95°C, 40 cycles of 95°C for 15 s, followed by 60°C for 30 s, followed by melt curve analysis. Technical triplicates of PCR reactions were performed. Primers were purchased from IDT DNA with the following sequences: EZH2_Fw (5’-TGCTTCCTACATCGTAAGTGCAA-3’); EZH2_Rv (5’-GGTGAGAGCAGCAGCAAACT-3’); MAPK14_Fw (5’-GTGGCCACTAGGTGGTACAG-3’); MAPK14_Rv (5’-GGGGTGTTCCTGTCAGACG-3’); SPRY2_Fw (5’-CCGCGATCACGGAGTTCA-3’), and SPRY2_Rv (5’-GACATGTACCTGCTGGGTGAG-3’).

### Plasmid Preparation

CMV-Luciferase-EF1α-copGFP (GFP-luc) lentiviral plasmid was purchased from BD Biosciences (BLIV511PA-1), and the lentiviral plasmid mCherry-Luc was a kind gift from Dr. Christian Badr (Massachusetts General Hospital). The miRH-Scr and miRH-124a encoding lentiviral vectors were purchased from SBI Biosystems. All plasmid constructs were propagated in Stbl3 (Thermo Fisher Scientific) or XL-1 Blue Ultracompetent bacteria (Agilent Technologies) on LB plates or in LB media with appropriate antibiotics. Plasmids were extracted by mini- or maxi-prep (Qiagen) following manufacturer’s recommendations. All cloned constructs were verified by Sanger sequencing prior to use.

#### Cloning of pTRIPZ-miR-Scr/miR-124a

The tet-inducible tRFP expression vector pTRIPZ vector was purchased from Dharmacon. The human sequence of pre-miR124.3 flanked with its 200 bp of genomic DNA upstream and downstream was custom designed by Gene Art with the synthetic addition of the XhoI/EcoRI restriction sites for subcloning into pTRIPZ vector in the miR30 context. Successful cloning was confirmed by colony PCR and plasmid sequencing (Macrogen). TRIPZ-infected cells were selected using puromycin (2 µg/ml).

#### miRNA Decoy Library Generation and Sequencing

The miRNA screen decoy library preparation and sequencing of GFP+ melanoma metastases were performed as described previously (25).

### Animal Studies

#### Mice

All experiments were conducted following protocols approved by the NYU Institutional Animal Care Use Committee (IACUC) (protocol number s16-00051). In this study, 6–8-week-old NOD/Shi-scid/IL-2Rgamma null [NOD.Cg-Prkdc^scid^Il2rg^tm1Wjl^/SzJ (NSG)] and athymic nude female mice were purchased from Jackson Laboratory (cat # 005557 and 002019, respectively) and maintained under standard pathogen-free conditions. When indicated, doxycycline hyclate (200 mg/kg/day) was administered to mice in food pellets.

#### Ultrasound-Guided Intracardiac Injection

For each mouse, 1 × 10^5^ cells suspended in 100 μl of PBS were injected by ultrasound guidance (Visualsonics Vevo 770 Ultrasound Imaging System) into the left ventricle of mice anesthetized with isoflurane. For some experiments, mice were monitored for metastatic progression by *in vivo* bioluminescent imaging [*In Vivo* Imaging System (IVIS)]. Upon substantial weight loss and/or signs of distress (neurological signs, abnormal locomotion) in any experimental mouse, experimental endpoint was established. At experimental endpoint, all mice in all experimental groups were euthanized in a CO_2_ chamber. Organs harvested were imaged using a fluorescent dissecting scope (Leica), prior to fixation in 10% formalin for 48 h, and embedding in paraffin.

#### Bioluminescence

Fifteen minutes prior to imaging, D-Luciferin substrate (150 mg/kg body weight; Gold Biotechnologies) was administered to mice by intraperitoneal injection. Mice were anesthetized with isoflurane and imaged by IVIS Illumina instrument (PerkinElmer) for an automatically determined duration (1–120 s). Signal was quantified using Living Image software (Xenogen) by measurement of average luminescent flux (p/s/cm^2^/sr) in drawn brain and body regions of interest (ROIs). Data were plotted using GraphPad PRISM, and significance was determined by unpaired t test.

#### Survival Analyses

Mice were euthanized when their body weight dropped at or under 17 g or developed signs of discomfort or neurological disorders, whichever came first. The experiment was concluded when mice from the Dc-Scr group that remained alive at day 100 were euthanized.

### Analysis of miRNA-124a Expression in Clinical Samples

Primary human melanoma samples were collected at the time of surgery from 92 patients. Informed consent was obtained from all patients, and approval was acquired from the institutional review board (IACUC) of NYU School of Medicine (protocol #10362). miRNA expression profiling of FFPE-extracted RNA from primary melanomas was performed by Exiqon, Inc., using dual-color miRCURY™ LNA arrays. The quantified signals were background corrected and normalized using the global Lowess regression algorithm. Data are deposited in GEO (accession: GSE62372) (20).

### Statistical Analyses

Statistical analyses were performed with GraphPad Prism (GraphPad Software, Inc.) Data are presented as the mean ± SD. Significance was determined using unpaired Student’s t test, chi square test, Mantel–Cox test, or Breslow–Wilcoxon test, where appropriate. The statistical analyses were performed, and p values were indicated in each figure legend. p values are represented as *p < 0.05, **p < 0.01, ***p < 0.001, ****p < 0.0001, and *****p < 0.00001.

## Data Availability Statement

The raw data supporting the conclusions of this article will be made available by the authors without undue reservation.

## Ethics Statement

The animal study was reviewed and approved by IACUC.

## Author Contributions

All authors listed have made substantial, direct, and intellectual contribution to the work and approved it for publication.

## Funding

This work was supported by the National Institute of Health/National Cancer Institute (NIH/NCI) grants R01CA202027 (EH), R01CA243446 (EH), P01CA206980 (EH, MB, NET), R33CA182377 (BDB), and NYU Melanoma SPORE 5P50CA225450 (IO).

## Conflict of Interest

The authors declare that the research was conducted in the absence of any commercial or financial relationships that could be construed as a potential conflict of interest.

## Publisher’s Note

All claims expressed in this article are solely those of the authors and do not necessarily represent those of their affiliated organizations, or those of the publisher, the editors and the reviewers. Any product that may be evaluated in this article, or claim that may be made by its manufacturer, is not guaranteed or endorsed by the publisher.
